# Solid-State Characterization of Nevirapine

**DOI:** 10.4103/0250-474X.45401

**Published:** 2008

**Authors:** Mahua Sarkar, O. P. Perumal, R. Panchagnula

**Affiliations:** Department of Pharmaceutics, National Institute of Pharmaceutical Education and Research (NIPER) Sector 67, S. A. S Nagar-160 062, India; 1 Department of Pharmaceutical Sciences, College of Pharmacy, South Dakota State University, Box 2202C, Brookings, SD-57007, USA

**Keywords:** Desolvation, nevirapine, powder dissolution, solid state, solvates

## Abstract

The purpose of this investigation is to characterize nevirapine from commercial samples and samples crystallized from different solvents under various conditions. The solid-state behavior of nevirapine samples was investigated using a variety of complementary techniques such as microscopy (optical, polarized, hot stage microscopy), differential scanning calorimeter, thermogravimetric analysis, Fourier transform infrared spectroscopy and powder X-ray diffractometry. The commercial samples of nevirapine had the same polymorphic crystalline form with an anhedral crystal habit. Intrinsic dissolution of nevirapine was similar for both the commercial batches. Powder dissolution showed pH dependency, with maximum dissolution in acidic pH and there was no significant effect of particle size. The samples recrystallized from different solvent systems with varying polarity yielded different crystal habits. Stirring and degrees of supersaturation influenced the size and shape of the crystals. The recrystallized samples did not produce any new polymorphic form, but weak solvates with varying crystal habit were produced. Recrystallized samples showed differences in the x-ray diffractograms. However, all the samples had the same internal crystal lattice as revealed from their similar melting points and heat of fusion. The intrinsic dissolution rate of recrystallized samples was lower than the commercial sample. It was found that the compression pressure resulted in desolvation and partial conversion of the crystal form. After compression, the recrystallized samples showed similar x-ray diffractograms to the commercial sample. Amorphous form showed slightly higher aqueous solubility than the commercial crystalline form.

The development of a successful formulation is dependent on the physicochemical and physico-technical properties of the active pharmaceutical ingredient (API), which has a direct impact on the manufacturing processes, stability, and bioavailability of the final drug product^1^,^2^. Majority of solid drugs and dosage forms are prepared in the crystalline state, characterized by a regularly ordered lattice structure having definite shape, structure and physical properties^2^. Each polymorphic form displays unique physicochemical properties like solubility, melting point, stability, hygroscopicity, hardness, compressibility, density, optical and electrical properties and vapor pressure^3^–^5^. Moreover, solid-state properties have a profound impact solubility and stability of a drug, which in turn are key contributors towards bioavailability^6^. Amorphous solids have gained widespread importance as they have higher solubility, higher dissolution rate and better compressibility than the corresponding crystalline forms^7^. Standard pharmaceutical processes such as; freeze-drying and spray drying lead to the generation of amorphous systems, which are thermodynamically unstable and have a tendency to revert to the crystalline form on storage (devitrification)^8^,^9^. FDA's drug substance guidelines state that, when filing an ANDA, appropriate analytical procedures employed to detect polymorphs, solvates, or amorphous forms of the drug substance should be documented^10^.

The present investigation deals with the solid state characterization of nevirapine (NVP), which has poor water solubility. Anhydrous form of NVP is used in tablet formulations, while nevirapine hemihydrate is used in suspension formulation^11^. Although, the drug appears to be readily absorbed orally, NVP particularly at higher doses (>50 mg) exhibit characteristics of solubility rate-limited absorption with a resultant decrease in bioavailability^12^. NVP is also used in fixed dose combinations (FDCs) along with lamivudine and stavudine. In comparison to other drugs, NVP is a poorly soluble hydrophobic molecule which poses processing problems in the manufacture of FDCs^13^. The objective of this study is to characterize the solid state properties of NVP recrystallized from various solvents for improving the physico-mechanical and biopharmaceutic properties by generating alternate solid forms of the drug.

## MATERIALS AND METHODS

Two different batches of NVP were obtained from Ranbaxy Labs (Gurgaon, India). Solvents were either of HPLC or analytical grade, and all other chemicals used were of laboratory grade.

### Equilibrium solubility determination:

Equilibrium solubility of NVP was determined at 60° in various solvent systems (Tables 1 and 2) to determine the degree of supersaturation attainable in the crystallizing solvent. The samples were withdrawn after equilibration, filtered through a 0.45 μm membrane filter. The filtered samples were then diluted with 0.1 N HCl and the concentration was determined by measuring the absorbance at 313 nm in a UV-Spectrophotometer (Beckman, USA).

**TABLE 1:** SOLVENT SYSTEMS USED FOR RECRYSTALLIZATION OF NEVIRAPINE

Sample Code	Solvent system	Polarity Index^a^
NR-A	THF/EA	4.3
NR-B	EA	4.4
NR-C	THF/EtOH	4.7
NR-D	EtOH/EA	4.8
NR-E	Water/EtOH	7.7

a Polarity index was calculated using the formula φ= φ_1_x_1_ + φ_2_x_2_, where φ_1_ and φ_2_are the polarity indices of the solvents 1 and 2 respectively, x_1_ and x_2_ are the volume fractions of the solvents 1 and 2 respectively. All the binary solvent systems are 1:1 proportions. Key: EA- ethyl acetate; THF-tetrahydrofuran.

**TABLE 2:** DEGREE OF SUPERSATURATION OF NEVIRAPINE IN VARIOUS SOLVENT SYSTEMS

Sample Code	Nevirapine concentration (mg/ml) at 60°		
	Λ_1_	Λ_2_	Λ_3_
NR-A	79.45	83.95	88.15
NR-B	62.88	66.17	69.48
NR-C	71.68	75.45	79.22
NR-D	46.59	49.05	51.50
NR-E	48.27	50.81	53.35

Three degrees of supersaturation (Λ_1_, Λ_2_, Λ_3_) were chosen based on the solubility data at 60°. Λ_2_ was obtained experimentally from the solubility data, while Λ_1_ is 5% lower than Λ_2_ and Λ_3_ is 5% higher than Λ_2_. The cooling rates were either fast (10 ml/min) or slow (0.250 ml/min). The cooling rate was controlled by circulating cold water around the crystallization chamber.

### Recrystallization experiments:

Supersaturated solutions of nevirapine were prepared by dissolving excess drug in 10 ml of selected solvent systems at 60°. The solutions were filtered and allowed to dry at room temperature. The crystallization was carried out in various solvent systems, which varied in their polarity index (Table 1)^14^. Commercial samples of NVP were recrystallized in flat bottom glass vials using an in-house crystallizer. The degrees of supersaturation and rate of cooling that were investigated are listed in Table 2 for the five solvent systems. Solvents for recrystallization were heated to 60°, where all the samples dissolved completely and then cooled at two different rates with or without stirring. Cooling was achieved by controlling the flow of water in the external chamber surrounding the crystallizer. The crystals were harvested, filtered and dried for 24-48 h in a desiccator and stored in tightly closed containers.

### Generation of amorphous form of nevirapine:

NVP was melted at 245-250° and immediately quench cooled in liquid nitrogen. The sample was analyzed by DSC and pXRD. The solubility of the amorphous system was determined by saturation solubility studies in 0.1 N HCl.

### Optical and hot stage microscopy:

Melting points and physical changes were visually examined at 50 X magnification by hot stage microscopy (HSM). The study was carried out using Leica DMLP polarized microscope and Leica LMV hot stage (Leica, Heidelberg, Germany). The crystal habit of the commercial and recrystallized samples were viewed and photographed under light microscope at various magnifications (10, 40, and 63X). NVP sample was mounted in air and/or silicone oil, and heated from 25° to 250° at a rate of 10°/min.

### Differential scanning calorimetry (DSC):

DSC thermograms were recorded using Mettler, Toledo 821 DSC (Mettler Toledo AG, Greifensee, Switzerland). Both temperature axis and cell constant were calibrated using indium. Four- to six- milligrams of the powder samples were weighed and analyzed in pin holed aluminum pans. NVP samples were exposed to different heating rates of 5, 10 and 20°/min over a temperature range of 25-500° under continuous nitrogen purging (80 ml/min).

### Thermogravimetric analysis (TGA):

TGA was performed using Mettler Toledo 851^e^ TGA/SDTA (Mettler Toledo AG, Greifensee, Switzerland). The loss of solvent/water was determined by heating the sample in aluminium crucibles at the rate of 10°/min from 25-500° under continuous nitrogen purge (20 ml/min).

### Fourier transform infrared (FT-IR) spectroscopy:

FT-IR spectra for NVP samples were recorded using FT-IR spectrophotometer (Perkin Elmer, Wellesley, MA, USA) equipped with Spectrum-1 analyzing software. Potassium bromide (KBr) disk pelletization method was employed for sample preparation method. Pellet was prepared by mixing 2-5% of the sample with potassium bromide in a mortar and compressed at a pressure of 15,000 psi. Each spectrum was derived from 16 single averaged scans collected in the mid IR region of 400-4000 cm^−1^ at a spectral resolution of 2 cm^−1^.

### Powder X-ray diffractometry (pXRD):

Powder X-ray diffraction patterns of different NVP samples were recorded at room temperature using a Brucker AXS X-ray diffractometer (Madison, WI, USA) configured with an online recorder. Radiations generated from Cu Kα source and filtered through Ni filters with a wavelength of 0.154 nm at 30 mA and 40 kV were used to study the X-ray diffraction patterns. The instrument was operated over the 2θ range of 5-60°. The experimental data comprising of 2θ and d-values of X-ray lines of the compound of interest were acquired by D8 Adjust software, which is configured to the instrument.

### Powder dissolution measurements:

The method described by Henwood *et al.* was used for powder dissolution studies^15^. Powder (100 mg) was first dispersed with sufficient quantity of glass beads (about 4 g) in a minimum amount of the dissolution medium. To this, 900 ml of media was added. Dissolution was performed in the compendial medium, 0.1 N HCl maintained at 37±0.5° in USP type II apparatus at 100 rpm. Samples were withdrawn at specific time intervals of 5, 10, 15, 20, 30, 40, 50 and 60 min, filtered through 0.4 μm HV filters and were quantified by UV-spectrophotometry at 313 nm. For commercial sample, the dissolution was also performed in 0.01 N HCl, pH 6.8 and pH 7.4. To study the effect of particle size on dissolution, NVP commercial samples were fractionated into coarse and fine fractions as defined in USP. Samples were fractionated through British standard sieves (BSS) with mesh number #36 (>355 μm), #52 (>180 μm) and #120 (125 μm), to obtain fractions of particle size >1000 μm (coarse) and less than 125 μm (fine). Dissolution study was carried out with each of these fractions.

### Intrinsic dissolution rate:

Intrinsic dissolution of commercial and recrystallized NVP was carried out in an Electrolab rotating disk USP type II apparatus (Mumbai, India) using 0.1 N HCl as the dissolution medium. The surface area of the drug pellet was kept constant at 50.24 mm^2^ and the pellet was prepared by compressing 100 mg of the drug in a hydraulic press at two different compression pressures, i.e. 1000 and 2000 psi for one minute. The compression pressure was optimized with the commercial bulk powder to avoid disintegration during the dissolution study. Base platform attached to the die was removed and screwed into a stainless steel hollow cylindrical holder, so that only one surface of the pellet is exposed to the dissolution medium. Dissolution was performed in 900 ml of 0.1 N HCl maintained at 37±0.5° at an agitation speed of 100 rpm. Precautions were taken to avoid entrapment of air bubbles on the surface of the drug pellet, which may decrease the effective surface for dissolution. Samples were withdrawn at regular intervals for 120 min (time to reach 10% of saturation solubility), which were then analyzed spectrophotometrically at 313 nm. The IDR was calculated as the cumulative release of drug per unit time per unit exposed surface area of the pellet.

## RESULTS AND DISCUSSION

**Fig. 1:**
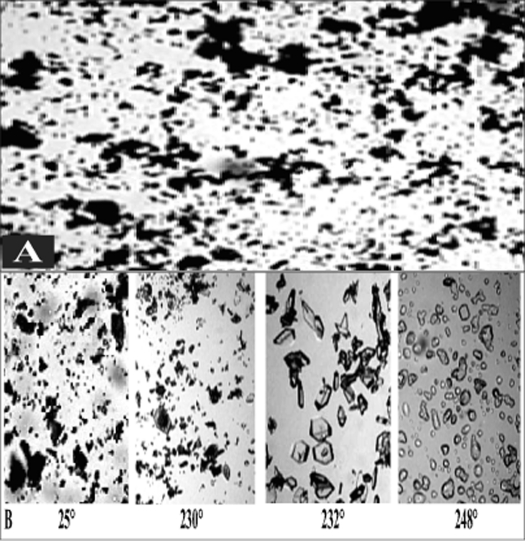
Optical and hot stage microscopy images of nevirapine sample (A) Optical microscopy and (B) hot stage microscopy images of commercial nevirapine sample. Optical microscopy images (63X) show aggregates of anhedral crystals. In hot stage microscopy, the aggregates under complete rearrangement into platy crystals at 230-235°, which finally melted at 248°.

**Fig. 2:**
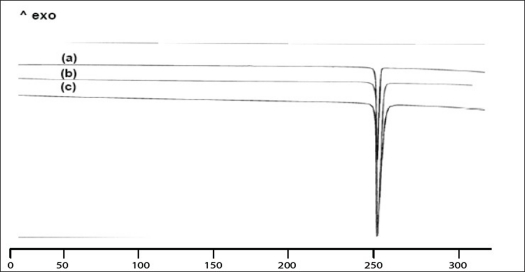
DSC thermograms of commercial sample of nevirapine DSC thermograms of commercial sample of nevirapine at different heating rates of (a) 5, (b) 10 and (c) 20°/min. Samples were heated from ambient to 300°.

**Fig. 3:**
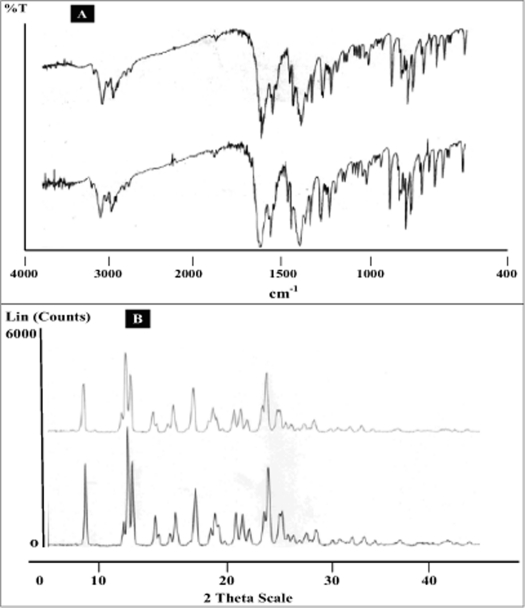
FT-IR spectra and Powder XRD difractograms of nevirapine A. FT-IR spectra and B. Powder XRD diffractograms of two different commercial batches of nevirapine.

Two batches of commercial sample of NVP was characterized as received by microscopy (optical, polarized and hot stage), DSC, TGA, FT-IR, pXRD and dissolution studies (intrinsic and powder dissolution). Morphological features of the NVP sample was examined by optical microscopy at 63X (fig. 1a). Powder consisted of irregularly shaped aggregates (anhedral or allotriomorphic crystals) at room temperature with the absence of birefringence under polarized light. Thermal events of the NVP sample were also determined with HSM. The powder agglomerates underwent complete reorganization at about 230-240° to form platy crystals, with good birefringence pattern, which finally melted at 248-250° (fig. 1b). No desolvation event was found when the sample was heated in silicone oil. NVP can exist either as anhydrous or hemihydrate form. During manufacture, hemi-hydrate form is crystallized which is dried either at low temperature (35-40°) to get hemi-hydrate or at high temperature (90-100°) to get anhydrous form^16^. The hemi-hydrate form is reported to exist as prismatic crystals, while the anhydrous form is found as fine-grained opaque aggregates in polarized microscope^16^. DSC analysis of NVP (fig. 2) showed a single endothermic fusion in the temperature range of 244.5-250.3° (ΔH_f_ = −147.8 J/g) with no polymorphic transitions. There was no significant change in enthalpy of fusion at different heating rates. When compared with the DSC curves, the TGA curves confirmed the endothermic event as melting, hence indicating the absence of any hydrate/solvate. The melting point found from HSM is consistent with DSC results and matches with the reported melting point of NVP^17^. FT-IR spectra of both the batches of NVP (fig. 3a) showed characteristic C-O stretching vibration of cyclic amide at 1646 cm^−1^, N-H and C-N stretch of 7-membered ring at 3295-3188 cm^−1^. A three dimensional regular arrangement of building units of NVP anhedral crystals was confirmed by pXRD. Fig. 3b shows the overlaid pXRD pattern for the two different batches of NVP. The powder shows sharp diffraction peaks (scattering angles 2θ at 13.1°, 13.6°, 25.5° and 9.3° with relative intensities of 100, 71, 70 and 68%, respectively). The similarity in both the patterns confirmed polymorphic sameness of the two different commercial batches.

**Fig. 4:**
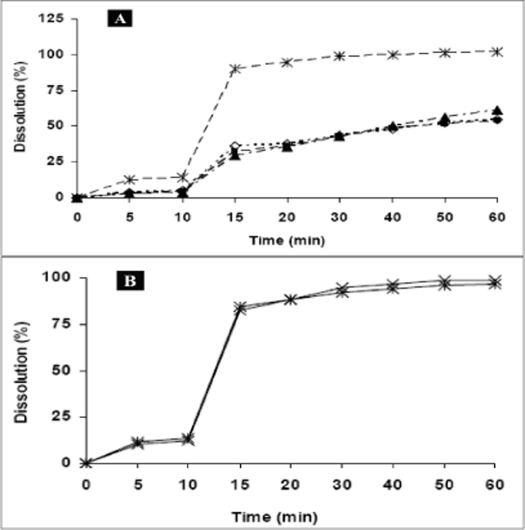
Powder dissolution of commercial samples of NVP under different conditions A. Powder dissolution of commercial sample of NVP at pH 1.2 (0.1 N HCl, –*–), pH 2.0 (0.01 N HCl, –▲–), pH 6.8 (–■–) and 7.4 (phosphate buffer, –◊–). All the determinations are mean of three experiments. Powder dissolution was conducted using paddle apparatus at 37° at 100 rpm B. Powder dissolution of coarse (–x–) and fine (–*–) fractions of commercial samples of NVP at 37° in 0.1N HCl at 100 rpm. The fractions were passed through BSS sieves with mesh number # 36 (>355-1000 μm) and # 120 (125 μm) to obtain the coarse and fine fractions respectively. No significant difference (p<0.05) was observed in the dissolution between these fractions.

Powder dissolution of NVP commercial samples was performed to determine the rate of drug release from simple powder at different physiological pH ranges. Maximum dissolution, i.e. more than 80% release was found in 0.1 N HCl (pH 1.2) and at higher pH values (pH 6.8 and 7.4) the release was less than 70% (fig. 4a). The drug is weakly basic (pKa= 2.8) and exhibits pH dependent solubility^13^,^18^. At pH values less than the pKa, it is highly soluble in aqueous buffer. As pH is increased the extent of ionization reduces and hence the amount released also declined. According to the Noyes-Whitney equation, for drugs with low aqueous solubility, particle size and the resulting surface area could have a significant effect on the rate of dissolution over the time interval during which gastrointestinal absorption occurs, and can affect the bioavailability^19^. Hence, to determine the influence of particle size on powder dissolution, the commercial samples were fractionated into coarse and fine particles and were subjected to dissolution in 0.1 N HCl at 100 rpm at 37±0.5°. The *f*2 value between the powder dissolution of coarse and fine fractions was calculated to be 86.45, which indicate that there is no significant effect of particle size on NVP dissolution (fig. 4b). Although, in general the decrease in particle size increases dissolution, poor wettability, agglomeration and/or development of electrostatic charges can preclude such an effect^20^.

IDR is defined as rate of dissolution of pure pharmaceutical ingredient when conditions such as surface area, agitation rate, pH and ionic strength of the dissolution medium are kept constant^21^. The study was carried out in 0.1 N HCl (pH 1.2) at two different compression pressures of 1000 and 2000 psi. The IDR constant was calculated as 0.087±0.007 and 0.088±0.006 μg/sq.mm/min at 1000 and 2000 psi, respectively. The difference in intrinsic dissolution rate at different compression pressures was not significant (p<0.005). Yu *et al.*^22^ suggested that IDR can closely correlate to the *in vivo* dissolution rate of a drug in comparison to powder dissolution. They proposed 0.1 mg/min/cm^2^ as the cut-off value to classify a drug as either highly soluble (Class I) or poorly soluble (Class II) according to the biopharmaceutic classification system. NVP is well below the specified value and hence has a tendency to show dissolution limited absorption *in vivo*.

Crystallization is a major technological process for particle formation in pharmaceutical industry. It affects most of the solid-state properties (particle size distribution, physical and chemical stability, structural and mechanical properties), which have direct implications on drug substance and drug product specific characteristics^23^. One of the most widely used methods for polymorph screening is crystallization from different solvents. Minor changes in crystallization conditions, for example, degree of supersaturation, temperature, presence of impurities and cooling rate can have a significant impact on the crystal and powder properties, notably on particle size, shape, and on the formation of defect structures^24^.

Solvent systems of varying polarity that are most likely to be encountered during manufacturing and processing were selected. The degree of supersaturation in different solvent systems was selected based on the solubility data. Binary systems allow the study of the influence of both polarity and nature of the solvent mixture^25^. We used binary solvent systems consisting of water, ethanol, tetrahydrofuran (THF) and ethyl acetate. These solvents cover a cover a relatively wide solubility parameter range^14^ (Table 1).

**Fig. 5:**
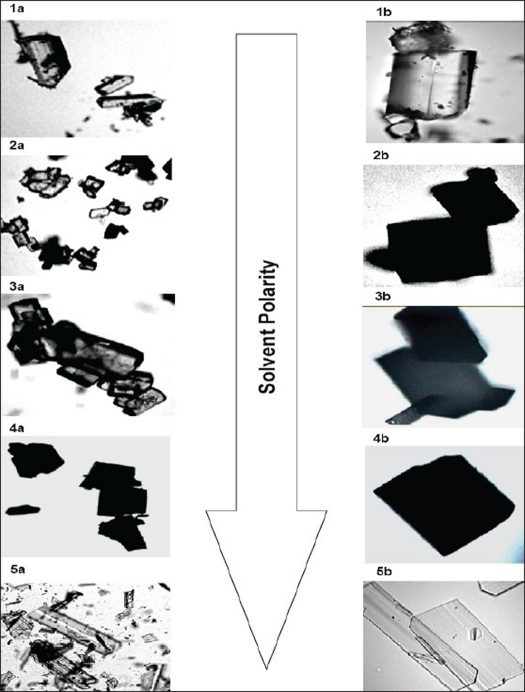
Microphotographs of NVP recrystallized from solvents of increasing polarity Microphotographs of NVP recrystallized from solvents of increasing polarity including THF/EA (1a and 1b), EA (2a and 2b), THF/E (3a and 3b), EA/E (4a and 4b) and Water/E (5a and 5b) showing the effect of solvent on crystal habit modification. Pictures were photographed at two different magnifications (10 and 63 X) represented by suffix a and b. E: ethanol; EA: ethyl acetate and THF: tetrahydrofuran

The crystal habit of NVP was strongly dependent upon the polarity of the crystallizing solvent. NVP crystals obtained from THF/EA (NR-A), EA (NR-B) THF/EtOH (NR-C) and EA/EtOH (NR-D) appeared as tabular and platy crystals (fig. 5). In highly polar water/ethanol (NR-E) system, elongated crystals were produced. These elongated crystals showed good birefringence. Depending on their polarity, solvents can preferentially interact with different crystal faces. It is suggested that polar solvents are preferentially adsorbed by polar faces and non-polar solvents by non-polar faces through the exposed functional groups^26^. If the solvent is strongly bonded to the solute at specific crystal face by interacting with specific functional groups, crystal growth would be rate limited by the removal of solvent from that face. As a result, the bonded surface grows slowly leading to a more elongated crystals^27^. Thus, NR-B with polarity index of 7.7 had higher affinity for the functional groups of NVP and resulted in elongated crystals. On the other hand platy crystals are formed when there is a weak solute-solvent interaction^26^, as is the case with the other solvent systems used in this study.

**Fig. 6:**
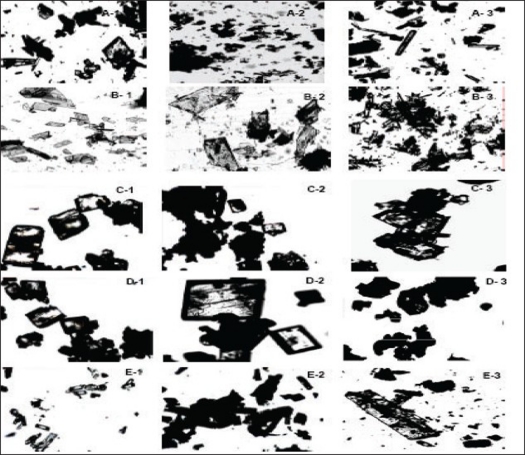
Microphotographs of NVP crystallized from different solvents Microphotographs of NVP crystallized from different solvents, A (THF/EA), B (EA), C (THF/E), D (EA/E) and E (Water/E), showing the effect of degree of supersaturation on crystal habit modification. Photographs were taken at 10X magnification and suffixes 1, 2, and 3 represent the increasing degree of supersaturation, where 1 and 3 are 5% lower and higher concentrated solution than 2, which is a supersaturated solution.

Within the same solvent system, many factors are known to influence the crystal habit including the initial supersaturation, cooling rate and agitation rate^26^. It was observed that crystal habit varied with the degree of supersaturation (fig. 6). The difference between concentration of the supersaturated solution, c, and the saturated concentration, c*, is the so-called absolute supersaturation, which is the driving force for the crystallization process and is given by the equation^28^: Δc = c − c*

At a constant temperature, supersaturation ratio and interfacial energy are key-factors that influence the nucleation rate. As the supersaturation is increased, the rate of nuclei formation is greater than crystal growth and growth occurs mainly in one direction, producing elongated crystals^26^. On the other hand, when there is a lesser degree of supersaturation, the solute-solvent interactions are insignificant, producing platy crystals.

Similarly, the rate of cooling alters crystal habit by its influence on degree of supersaturation. Crystallization at a slower cooling rate produced more symmetric crystals compared with faster cooling rate, where generally more asymmetric thin plate like crystals are formed. Crystals obtained with faster cooling rate were smaller in comparison to slower cooling rate (data not shown). Rapid cooling a supersaturated solution reduces solubility and results in rapid deposition of drug molecules on the nuclei. Nucleation is faster than crystal growth rate during faster cooling therefore; many small crystals appear instead of few crystals growing to sufficiently larger size^29^.

Agitation has an important effect on the process of crystallization. The aspect ratio (ratio of horizontal maximum and vertical maximum distance of the particle) was highest for unstirred conditions than during stirring. Rapid stirring caused even distribution of crystallizing solute on the nuclei, resulting in elongated crystals with narrow particle size distribution (data not shown). The crystals obtained under stirring conditions were fine crystals since stirring facilitates the rate of nucleation by an even distribution of the solute molecules in the solvent. Increase in nucleation rate is the result of collision of initial crystals with the stirrer and formation of smaller seeds for further crystallization^30^. Additionally, stirring also break larger crystals into smaller ones. Large platy crystals were produced under unstirred conditions as molecules from supersaturated solution deposit on selected crystals. External appearance of a crystal can thus be altered by changing the growth environment to suit the requirements. The solvent of crystallization tends to affect the drug crystal properties in variable ways making it difficult to predict the crystal habit in a particular solvent for a certain chemical class of drug. Recrystallization of NVP from all solvents by cooling method yielded the same polymorphic form as was evidenced from DSC, FTIR and pXRD results.

**Fig. 7:**
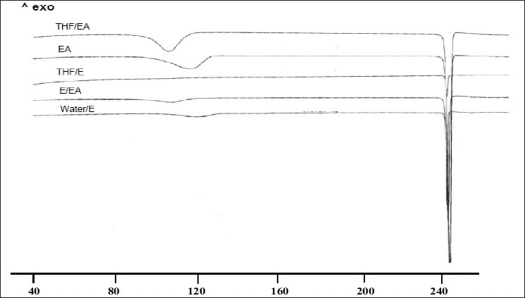
DSC traces of untreated and recrystallized nevirapine commercial sample DSC traces of untreated nevirapine commercial sample along with the recrystallized products from solvent systems arranged in increasing order of polarity from top to bottom. THF: tetrahydrofuran; EA: ethyl acetate and E: ethanol

**Fig. 8:**
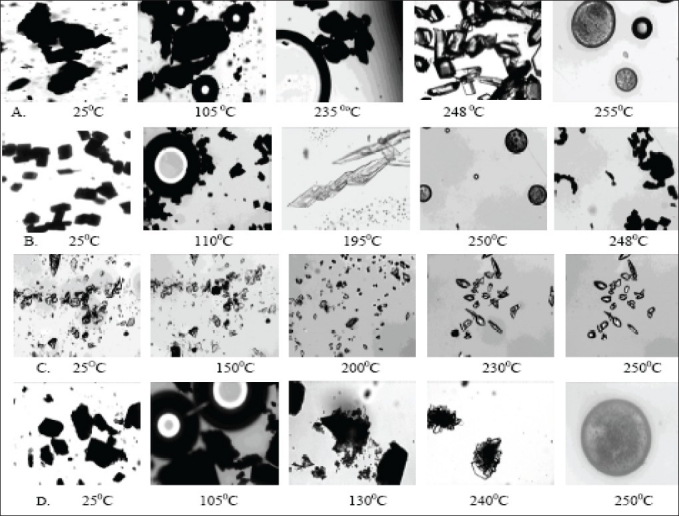
Hot stage microscopy microphotographs of recrystallized forms of Nevirapine Recrystallized samples were heated in silicone oil and the thermal events were observed at 50X magnification. NR-A (THF/ethyl acetate), NR-B (ethyl acetate), NR-D (ethanol/ethyl acetate) and NR-E (water/ethanol) undergo desolvation, followed by generation of micro crystals on the surface of larger crystals, which finally melt at 250-255°. On cooling the NR-B melt to 195°, it recrystallized into long chain of prismatic crystals, which were strongly birefringent. NR-C (THF/ethanol) did not show loss of any solvent molecule, but recrystallization at above 230° was evident (as in the case of commercial sample), which finally melted at 250°.

DSC thermogram of samples recrystallized from EA/EtOH, water/EtOH, THF/EA and EA showed two endotherms (fig. 7). The first broad endotherm was attributed to desolvation based on the evidence provided by TGA (results not shown) and HSM (fig. 8) followed by a sharp melting endotherm at 245°. Recrystallized samples from THF/EtOH showed only one sharp melting endotherm at 245°, which corresponded to drug melting. The fusion enthalpies (ΔH_f_) for melting endotherm varied from 130-140 J/g (Table 3) for various samples and the difference was not statistically significant (p<0.05). The sample crystallized from EA showed the least enthalpy of fusion (121 J/g) in comparison to other samples. However all the samples melted at the same temperature (245-246°), indicating that they all had the same crystal lattice. It is seen from the literature that the heat of fusion of crystalline form can be affected by the particle size and the crystal defects in the sample, although they belong to the same polymorphic form^31^,^32^.

**TABLE 3:** DSC DATA OF RECRYSTALLIZED NEVIRAPINE SAMPLES

Sample Code	1^st^ endotherm	Enthalpy (J/g)	2^nd^ endotherm	Enthalpy of fusion (J/g)
NVP-CS	-	-	244.94±0.14	145.22±0.77
NR-A	102.57±1.57	51.85±1.11	245.23±0.08	131.52±1.31
NR-B	98.19±2.79	34.07±3.02	246.04±0.14	120.98±2.59
NR-C	-	-	245.81±0.76	138.80±1.56
NR-D	106.95±4.20	51.77±5.54	245.37±0.19	131.70±1.13
NR-E	122.60±2.60	66.36±8.71	245.23±0.13	136.19±5.79

The sample codes represent the solvent systems from which NVP was recrystallized. NR-A is ethyl acetate/ ethanol (1:1); NR-B is ethyl acetate; NR-C is THF/ethanol (1:1); NR-D is ethyl acetate/ethanol (1:1) and NR-E is water/ethanol (1:1). NVP-CS is the commercial sample and analyzed as received, without any processing. The temperatures and enthalpy values are given as mean±SD. The first endotherm denotes the temperature of solvent removal from the sample. NR-E does not undergo desolvation. There is no significant difference (*P*<0.05) in melting points (second endotherm) and enthalpy values of the commercial and recrystallized samples.

To characterize the first endotherm in the recrystallized samples, TGA, first derivative TGA and DTA (Differential thermal analysis) were recorded simultaneously. The TGA thermograms of NR-A, NR-B, NR-D and NR-E showed 8.3, 6.4 2.5 and 2.2% loss in weight at temperatures corresponding to 103.1, 103.7, 93.6 and 123.1°, respectively. These desolvation temperatures depend on the boiling point of single/binary solvent system from which the samples were recrystallized. The first derivative TGA gives a measure of weight loss at a particular temperature to the total initial weight. DTA of all the samples showed an endotherm corresponding to the melting temperature of NVP at 250°. The weight loss from TGA was negligible (less than 0.4 moles of solvent) compared to the theoretical weight loss expected for a stoichometric solvate. Furthermore the desolvation event was substantiated from HSM. On heating, the samples (NR-A, NR-B, NR-D and NR-E) clearly showed loss of solvent molecules from the crystal surface (fig. 8). NR-C did not show any desolvation event. Desolvation from the crystal lattice occurred in the range of 80-110° for all the crystal forms, depending on the boiling point of the solvent mixtures and the extent of binding of the solvent molecule to the crystal. As the solvent was released, the crystals became opaque with simultaneous change in the dimensions of the crystals. Small crystals grew over the irregularly shaped masses in the powder, which gave good birefringence pattern in polarized light. In case of NR-C, highly polarized platy crystals were formed above 240°, which finally melted at 250°. From the results, it appears that the solvent was weakly adsorbed to the samples and did not form a definite stoichometric ratio within the crystal lattice. NVP is known to exist as a hemi-hydrate when crystallized from an aqueous solution^33^, however, the results from our study (water/EtOH) did not produce a hemi-hydrate (TGA weight loss corresponds to <0.25 moles of water). The difference can be attributed to the difference in the water activity in the crystallizing solvent which influences the formation of hydrate of definite stoichoimetry^34^.

Major absorption bands of C=O stretch and N-H stretch of cyclic amide in NVP molecule appeared in the FTIR spectra of all recrystallized samples. This indicated that there was no difference in the internal structure and conformation of these samples. Since polymorphs and solvates differ from each other mainly with respect to hydrogen bonding, the spectral differences in NVP at characteristic region due to stretching of functional groups like C=O, N-H can be attributed to interaction of the adsorbed solvates. The sample recrystallized from water/EtOH showed a prominent peak at 3500 cm^−1^ which can be attributed to the O-H stretching of water. Samples recrystallized from solvents containing ethyl acetate showed sharp ester stretching vibrations from at 1740 cm^−1^.

**Fig. 9:**
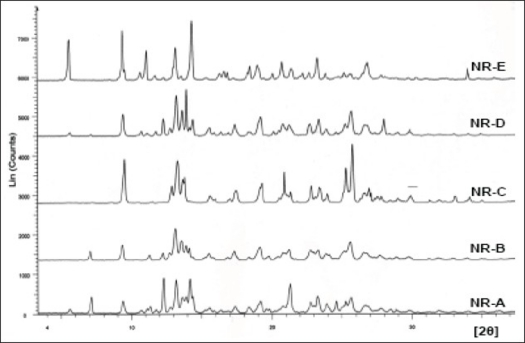
Powder XRD patterns of crystallized nevirapine samples from various solvent systems Powder XRD patterns of nevirapine samples crystallized from water/EtOH (NR-E), EA/E (NR-D), THF/E (NR-C), EA (NR-B) and THF/EA (NR-A) in the decreasing order of solvent polarity (top to bottom). E: ethanol; EA: ethyl acetate and THF: tetrahydrofuran

The pXRD patterns of all the recrystallized products (NR-E, NR-D, NR-C, NR-B and NR-A) are shown in fig. 9 in the order of decreasing solvent polarity. The characteristic d-values and relative intensities (I/I_o_) of the major peaks were compared with those from the commercial sample (Table 4). As the polarity of the solvent system was increased, the pattern in the 12-16° 2θ region became more ordered (peak clusters were well separated and resolved) representing a gradual increase in degree of crystallinity. In NR-E, a strong X-ray line at 5.54° 2θ was observed, which was absent in the commercial sample. Two solid forms are said to be different from each other if their scattering angles of ten strongest reflections differ by ±0.20° and relative intensities of these reflections vary by more than 20%^35^. The relative intensity of peaks varied in all samples and these variations were attributed to differences in crystal habit and size that resulted in preferred orientation of the crystals in the sample holder^34^. However, further studies are required to explain the differences in the pXRD patterns observed with some of the recrystallized samples.

**TABLE 4:** COMPARISON OF SOME MAJOR XRD PEAKS OF COMMERCIAL AND RECRYSTALLIZED NEVIRAPINE SAMPLES

Samples	2θ	I/I_o_	2θ	I/I_o_	2θ	I/I_o_	2θ	I/I_o_
NVP-CS	13.1	100	13.6	71	-	-	15.4	25
NR-A	13.1	94	13.5	47	14.1	99	-	-
NR-B	13.1	100	13.5	61	13.8	46	15.4	21
NR-C	13.2	74	13.7	42	-	-	15.6	13
NR-D	13.2	86	13.6	54	13.9	100	15.5	17
NR-E	13.1	58	-	-	14.3	100	-	-
Compressed^a^ samples							
NR-A	13.2	100	13.5	57	14.3	43	15.5	18
NR-B	13.2	100	13.6	63	-	-	15.5	21
NR-C	13.2	100	13.6	69	-	-	15.5	21
NR-D	13.2	100	13.6	62	-	-	15.5	17
NR-E	13.2	66	13.6	23	14.4	100	15.5	9

a The pellets were compressed at 1000 psi in a hydraulic press. The pellets on compression show a XRD which resembles more of the commercial sample.

**Fig. 10:**
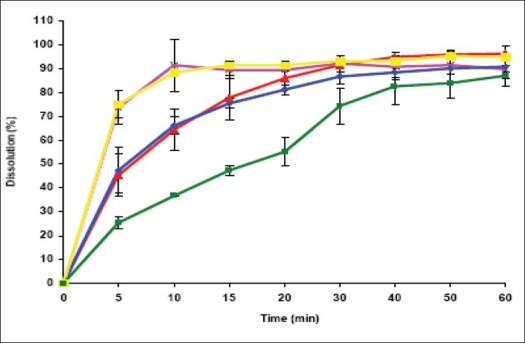
Dissolution profiles of nevirapine samples obtained from different solvent systems The dissolution profiles of nevirapine samples obtained from different solvent systems. The dissolution was performed in 0.1 N HCl. NR-A is NVP crystallized from ethyl acetate/ethanol (1:1) (
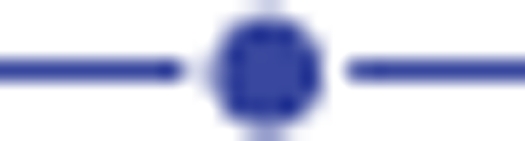
); NR-B: water/ethanol (1:1) (
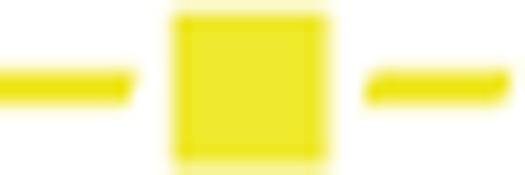
); NR-C: THF/ethyl acetate (1:1) (
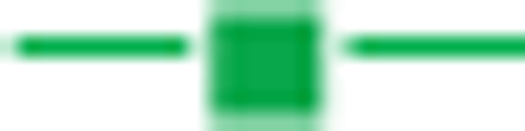
); NR-D:100% ethyl acetate (
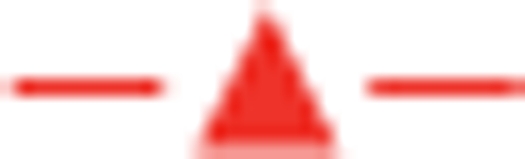
) and NR-E:THF/ethanol (1:1) (
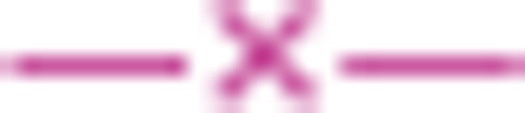
). The data is shown as mean±SD, (n=3).

The dissolution profiles of NVP recrystallized from different solvents are shown in fig. 10. In the first 10 min, the extent of dissolution was highest for NR-B and NR-E (>80%), followed by NR-A and NR-D (>50%), while NR-C showed the least dissolution. The initial rate of dissolution of the recrystallized products is significantly higher than that of the commercially sample (fig 4a and 10. Though the overall extent of dissolution was found to be similar in all recrystallized samples, the rate of dissolution was found to vary significantly for all the samples. The amount of drug dissolved depends on the size and number crystal faces exposed to the dissolution medium and the outer face of solid is influenced by liquid from which it is crystallized. Furthermore, the difference in dissolution rate is often related to the particle size and surface area of various crystals exposed to the dissolution medium^37^.

**Fig. 11:**
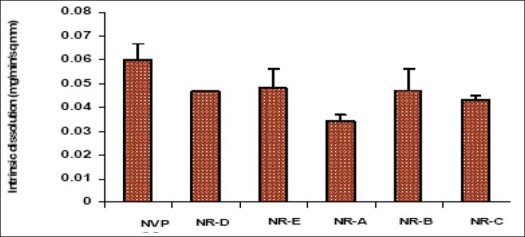
Intrinsic dissolution rates of different nevirapine samples at pH 1.2 in 0.1 N HCl Intrinsic dissolution rates of different nevirapine samples determined at pH 1.2 (0.1N HCl). NVP-CS is the untreated commercial sample of nevirapine. NRA-E are samples recrystallized from various solvent systems. There is difference in the IDR of commercial and the recrystallized samples. The data is shown as mean with SD for a sample size of n=3.

Intrinsic dissolution rate of the recrystallized samples was determined in 0.1 N HCl by exposing a constant surface area of the pellet to the dissolution medium. It was observed that there was significant difference in the IDR of commercial and the recrystallized samples (fig. 11). The IDR of various solvates was 20-43% lesser than the commercial samples, but there was no significant difference among the recrystallized samples except NR-A. Sample recrystallized from the least polar solvent showed the lowest IDR (THF/EA). This sample also had a higher solvate adsorption as evidenced from the TGA results. The observed difference in IDR can be ascribed to the altered crystallinity^38^, i.e., crystal defect, which is in agreement with the DSC and XRD results. Moreover, poor wettability of the pellet in the aqueous medium due to the adsorbed solvent could also have decreased the IDR. pXRD pattern of the compressed pellets of the recrystallized samples showed difference in spectra in the 12-16° 2θ values, when compared to their original spectra of (Table 4). This suggested a partial transformation of the solid state form possibly by desolvation during compression. The diffractograms of the compressed samples were more similar to the commercial sample. Detailed studies are warranted to comment on this compression induced transformation.

**Fig. 12:**
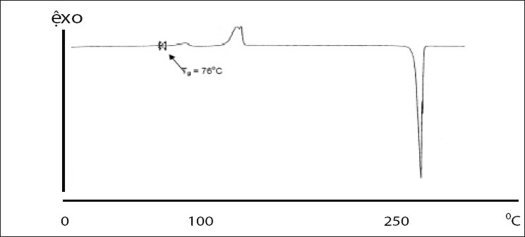
DSC scans of the amorphous form of NVP prepared by quench cooling method DSC scans of the amorphous form of NVP prepared by quench cooling method. Powder samples of 8-10 mg were analyzed under dry nitrogen atmosphere (80 ml/min) in pin holed aluminium pans at a heating rate of 50°/min. The amorphous form shows glass transition temperature at 76°, followed by recrystallization at 129° to the stable crystalline form which melts at 245°.

Amorphous form of NVP was generated by quench cooling the melt of commercial sample. The sample showed the absence of long range order with no definite crystal structure. Particle shape was undefined and glass like with smooth edges (data not shown). In HSM, volume changes were observed with the gradual increase in temperature. As the temperature was increased above 70°, the powder mass became opaque. On further raising the temperature above 120°, the powder mass arranged into anhedral crystals, similar to the commercial sample. Above 200°, the anhedral crystals were observed to change into platy crystals which melted in the narrow range of 245-250° (data not shown). DSC thermogram of amorphous NVP (fig. 12) showed a glass transition onset at 76.04±0.29° followed by crystallization exotherm at 129.66±0.77° (ΔH_c_ =42.49±2.5 J/g) and a final melting endotherm at 245.30±0.19° (ΔH_f_ =142.41±0.81). The thermal events were in agreement to the observations in HSM. The glass transition and recrystallization events observed in DSC prove the amorphous nature of the quench cooled product. Glass transition temperature, at which the molecular mobility is restricted, is a characteristic of amorphous systems and helps to distinguish between the amorphous and microcrystalline states^7^. Conclusive evidence of generation of amorphous form was provided by pXRD. The crystalline form showed characteristic diffraction peaks at different angles, while the amorphous form shows a halo, with no discernible peaks.

**Fig. 13:**
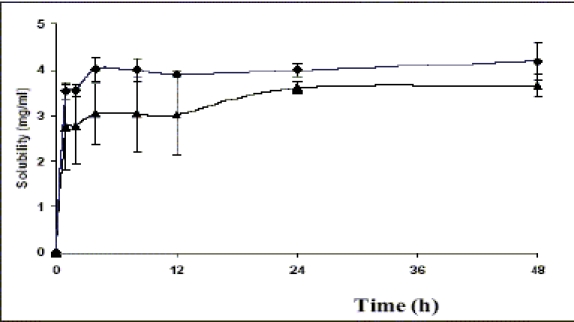
Solubility of amorphous and crystalline form of nevirapine Solubility of amorphous (–◆–) and crystalline (–▲–) form of nevirapine at 37° in 0.1N HCl. The data is shown as mean±SD, (n=3).

Solubility of the amorphous form was found to be slightly more than the crystalline form. The peak solubility of the amorphous form was seen within 4 h of the start of experiment and there was no further increase in solubility at later time points. (fig. 13). The saturation solubility was found to be 4.32±0.09 mg/ml in 0.1 N HCl after 48 h of the experiment, which was 18% higher than that of the commercial crystalline form. The amorphous form being the high energy state, due to lack of long range order, produces a greater molecular mobility and thermodynamic escaping tendency leading to faster and higher solubility^8^. However, the lower magnitude of increase in solubility of the amorphous powder was due to the poor wettability and the very electrostatic nature of the quench cooled product^39^. Although, the amorphous form was found to be sufficiently stable up to two weeks under refrigerated conditions, further studies are required to investigate the stability of the amorphous form at room temperature.

In conclusion crystallization of nevirapine under a variety of crystallizing conditions resulted in change in crystal habit of the drug without any change in the internal crystal lattice. The solvent used for crystallization was found to form weak solvates. Size of the crystals was significantly influenced by cooling rate and agitation rate. The particle size of the commercial sample did not show any significant influence on drug dissolution. On the other hand, recrystallized samples showed higher powder dissolution and lower IDR than the commercial sample. The amorphous form was more soluble than the commercial sample. Further studies are required on the amorphous form of the drug to improve the drug's solubility. Similarly, the crystal habit changes can have an influence in developing better solid dosage forms of nevirapine.
